# Deciphering the Composition and Functional Profile of the Microbial Communities in Chinese Moutai Liquor Starters

**DOI:** 10.3389/fmicb.2019.01540

**Published:** 2019-07-04

**Authors:** Shu-Heng Gan, Fan Yang, Sunil Kumar Sahu, Ru-Ye Luo, Shui-Lin Liao, He-Yu Wang, Tao Jin, Li Wang, Peng-Fan Zhang, Xin Liu, Jin Xu, Jing Xu, Ya-Yu Wang, Huan Liu

**Affiliations:** ^1^BGI-Shenzhen, Shenzhen, China; ^2^China National GeneBank, BGI-Shenzhen, Shenzhen, China; ^3^China Kweichow Moutai Distillery Co., Ltd., Zunyi, China; ^4^State Key Laboratory of Agricultural Genomics, BGI-Shenzhen, Shenzhen, China; ^5^BGI Education Center, University of Chinese Academy of Sciences, Shenzhen, China; ^6^Department of Biotechnology and Biomedicine, Technical University of Denmark, Copenhagen, Denmark

**Keywords:** Moutai liquor, fermentation starter, metagenomics, 16S rRNA gene, OTU, Daqu, saccharifying power, immature starter

## Abstract

Moutai is a world-famous traditional Chinese liquor with complex taste and aroma, which are considered to be strongly influenced by the quality of fermentation starters (Daqu). However, the role of microbial communities in the starters has not been fully understood. In this study, we revealed the microbial composition of 185 Moutai starter samples, covering three different types of starters across immature and mature phases, and functional gene composition of mature starter microbiome. Our results showed that microbial composition patterns of immature starters varied, but they eventually were similar and steady when they became mature starters, after half-year storage and subsequent mixing. To help identify two types of immature starters, we selected seven operational taxonomic unit (OTU) markers by leave-one-out cross validation (LOOCV) and an OTU classified as *Saccharopolyspora* was the most decisive one. For mature starters, we identified a total of 16 core OTUs, one of which annotated as *Bacillus* was found positively associated with saccharifying power. We also identified the functional gene and microbial composition in starch and cellulose hydrolysis pathways. Microbes with higher abundances of alpha-glucosidase, alpha-amylase, and glucoamylase probably contributed to high saccharifying power. Overall, this study reveals the features of Moutai starter microbial communities in different phases and improves understanding of the relationships between microbiota and functional properties of the starters.

## Introduction

Moutai (Maotai) liquor, one of the most recognized Chinese traditional liquors, is a crystal-clear Jiang (soy sauce) flavor distilled beverage (53 percent alcohol by volume). As the products of sorghum and wheat using solid-state fermentation, it is well-known for its complex pleasant aroma, containing more than one thousand volatile compounds. The rich and mellow flavor is enduring and the aftertaste is lasting. The only place producing authentic Moutai liquor is Moutai town (27°51′ N 106°22′ E, altitude 423 meters), located in the north of Guizhou Province in China. This small town is in the valley of Chishui River and the lowest point of Guizhou Plateau, with 800∼900 mm annual rainfall, annual 326 days without frost, 5 months of prolonged high temperature (35∼39°C), and 1400 h of sunshine a year (Miao, 2013). Producing around fifty thousand tons of Moutai base wine and generating 73.6 billion RMB (about 10.7 billion USD) in revenue in 2018, Kweichow Moutai Co., Ltd., the main Moutai liquor distillery, adheres to the traditional manufacturing processes to maintain consistency of the unique flavor. Over the past decades, the Moutai liquor industry has been trying to fulfill its burgeoning demand, but the efforts are almost unfeasible due to the geological constraints and other unknown factors. It is believed that the special microbial compositions contribute to its unique aromas and tastes. Uncovering the key microbes and their functions in the fermentation process will facilitate the full understanding of this traditional technique.

The manufacturing of Moutai liquor consists of two main processes: Daqu preparation and liquor fermentation. Daqu (big starter) is a brick-shaped solid-state fermentation starter culture named for its big size. Moutai Daqu is the largest and heaviest one of all (37 × 23 × 6.5 cm, 4.8–5.0 kg) (Zheng et al., 2011). The complex producing process of Daqu leads to its higher diversity of microbes and richer flavor than other kinds of simpler starters (Xiaoqu and Fuqu) (Zheng and Han, 2016; Wang et al., 2019). The Daqu-making process consists mainly of three parts: shaping, fermenting (about 1 month), and ripening (about 6 months) (Zhang, 2011). First, raw materials, including crushed wheat grains, few good mature starters (produced in the last round), and water, are mixed and pressed into brick shape (Supplementary Figure S1, “1. Mix raw materials” and “2. Shape”). Second, starter bricks are piled up with space between each two, covered with straw, and sprinkled with water, to create a proper fermentation environment for microbes; when the brick temperature reaches around 65°C, starter bricks are rearranged to adjust the temperature and humidity, letting each brick fermenting evenly (Supplementary Figure S1, “3. Stack” and “4. Rearrange”). After 2 weeks, when the brick temperature is close to room temperature, the workers dismantle starter brick walls and get phase I starter: immature starter (Supplementary Figure S1, “5. Dismantle”). Immature starter bricks can be separated into three types by color: yellow starter (well fermented, 80∼85%), white starter (under-fermented, 10∼15%), and black starter (over-fermented, <1%). Third, all immature starter bricks are stored for ripening in the open air for about 6 months, and then crushed and mixed (Supplementary Figure S1, “6. Store 6 months” and “7. Mix”). This mixture of powder is the phase II starter: mature starter. The following Moutai liquor fermentation is a process composed of repeated month-long cycles (Supplementary Figure S1 right dotted box). The repeated steps of the cycles are: steaming the materials, cooling down the distilled grains, mixing the grains and starter powder, piling up the grain-starter mixture for 4∼5 days (stacking fermentation), filling pits with the mixture and sealing them with mud for anaerobic fermentation for a month, taking out the fermented grains and steaming, and finally collecting the liquor. The raw material (crushed sorghum grains) is only added in the first two cycles while the starter powder is added in each cycle. The total weight of sorghum grains is the same as starter powder.

As microbes in starters are the one who actually performs fermentation, various methods have been applied to explore the microbial composition and activities in the liquor fermentation process. The classical microbiological methods, such as identification based on isolation and cultivation or PCR-DGGE, have offered early evidences of common microbes in the Chinese alcoholic fermentation (e.g., *Bacillus*, *Saccharomyces*, *Lactobacillus*, *Aspergillus*, and *Weissella*) (Wang et al., 2008, 2016; Zheng et al., 2011; Lv et al., 2012). However, these widely used methods are unable to discover the unculturable or low-abundant microbes and have a shortage of estimating the microbial diversity in breadth and depth. Recently, the high-throughput sequencing techniques have largely enhanced metagenomic researches by quickly generating a gigantic amount of sequence data covering a wide range of microbes in big samples size (Caporaso et al., 2011). The amplicon sequencing strategies, including 16S/18S rDNA and ITS sequencing, are convenient to capture the microbial diversity profile, while the metagenomic shotgun sequencing can provide the gene profile for functional analysis. These techniques have been widely applied to Chinese liquor studies, revealing the secrets behinds their traditional fermentation processes (Li et al., 2013; Xie et al., 2013; Guo et al., 2014; Tao et al., 2014; Zhang et al., 2014; Hong et al., 2016; Huang et al., 2017).

However, these metagenomic studies in Chinese liquor starters rarely used a large sample size and may lead to invalid arguments. For example, the understanding of microbial diversities in this special fermentation environment can be possibly misled by biased sampling. While the proportion of yellow starters in all immature starters is crucial to Moutai liquor quality, the difference of microbial communities between yellow starters and other immature starters has not been explored yet. In our previous study, we explored the microbial succession during the production of starters (Wang et al., 2015). Therefore, the present study was aimed to (1) expose the full scope of the microbial composition of Moutai starters, (2) identify the microbial signatures to distinguish between two types of immature starters, and (3) understand the functional relationship between the microbes and physiochemical properties of mature starters.

## Materials and Methods

### Sampling and DNA Extraction

We collected a total of 185 samples from three types of Moutai starters across two different phases in 2013. After about a month of fermentation, we got the brick-shaped phase I starters and named them “immature starters.” They could be divided into three types basically based on their color: yellow (Y, 1st type, *n* = 27), white (W, 2nd type, *n* = 27), and black (no sample) (Supplementary Figure S1). Then, immature starters were stored in the open air for half a year and their powder mixtures were the phase II starters, called “mature starters” (3rd type in our study, *n* = 131). In each Moutai liquor production cycle, a fresh batch of mature starters was produced for liquor fermentation. We took mature samples from six batches shortly after they were produced and named them B0∼B5 (B0, *n* = 26; B1, *n* = 24; B2, *n* = 4; B3, *n* = 32; B4, *n* = 30; B5, *n* = 15). As the black starters are extremely rare (usually less than 1% of total immature starters) and do not exist in some batches, we focused on the yellow and white immature starters besides mature starters in this study. The sample collection and DNA extraction were carried out according to the method described by Wang et al. (2015). The measurements of eight physicochemical properties, including acidity, reducing sugar fraction, moisture, starch fraction, saccharification (saccharifying power), liquefaction (liquefying power), protease activity (PA), and cellulase activity (CA), were conducted shortly after mature starter sampling. The detailed methodology is described in the Supplementary Material.

### 16S rRNA Amplicon Sequence Data

The extracted DNA was quantified in a Qubit fluorometer, and then all the 185 samples were amplified using primers 515F (5′-GTGCCAGCMGCCGCGG-3′) and 907R (5′-CCGTC AATTCMTTTRAGT-3′) for the V4-V5 region of 16S rDNA (Beller et al., 2012). The sequencing was performed by utilizing the Roche 454 GS FLX+ platform. The sequencing reads were trimmed and analyzed by Mothur (version 1.39.5) (Schloss et al., 2009) following the 454 SOP^1^ (except for the “Error Analysis,” accessed on November 2nd, 2017) (Schloss et al., 2011) to obtain the Operational Taxonomic Units (OTUs, 97% identity). The potentially chimeric sequences were identified and removed by “chimera.uchime” and “remove.seqs” of Mothur with “name” and “group” options using more abundant sequences in our samples as the reference. The rest reads were classified using RDP version 16, and the sequences belonging to “Chloroplasts” and “Mitochondria” class were removed and excluded from the study. The downstream analyses were performed with QIIME version 1.9.1 (Caporaso et al., 2010) and R version 3.4.3. The OTUs present in only one sample or with less than 20 tags were removed using the filter_otus_from_otu_table.py, and finally, 752 OTUs with a total count of 784362 sequences were selected after the screening. Alpha diversity values, including Shannon index, observed OTUs and phylogenetic diversity, were calculated with alpha_diversity.py at 988 sequences per sample (as 988 was the minimum sequence count) and plotted with R package “ggplot2” version 2.2.1. The Venn plots of three groups (Mature, White, and Yellow) in OTUs as well as in taxa were plotted with R package “eulerr” version 4.1.0. The Spearman correlation analysis of mature core OTUs (present in more than 95% mature samples) was calculated with function cor() from package “stats” and plotted using the “corrplot” version 0.84.

### Leave-One-Out Cross Validation With Random Forest Algorithm

To search the marker OTUs of immature starters, we performed leave-one-out cross validation (LOOCV) on a total of 54 immature samples with R (version 3.4.3). The process was: first, we left one sample as test data and used the rest 53 samples as training data to select OTU markers; next, a random forest classifier was built on training data with only the subset of OTU markers; then, the classifier was used to predict the left one sample, and the feature importance rank was kept; changed another sample as test data and repeated the steps above until all samples were tested. Analyzing the 54 lists of OTU markers and importance rank, we finally selected 7 OTU markers that occurred in more than 90% tests (≥49). The immature OTUs table was normalized in cumulative sum scaling (CSS) method with the R package “metagenomeSeq” 1.20.1 (Paulson et al., 2013). The feature selection step was done with R package “Boruta” version 6.0.0. The random forest classifier was built with R package “randomForest” 4.6-12 (Liaw and Wiener, 2002).

### Redundancy Analysis

To explore the relationship between microbial composition and physical and chemical properties of Moutai mature starters, we performed a redundancy analysis (RDA) of the total OTUs and eight physiochemical properties using the R package “Vegan” 2.4-6^2^. As there were too many OTUs, only the obvious relevant OTUs were displayed in the triplot. The permutation test was performed with the function “anova()” of R package “stats” 3.4.3.

### Metagenome Sequence Data and Gene Set

For the analysis of the functional genes, shotgun metagenome sequencing was performed for the five mature samples (B0.07, B0.22, B3.01, B4.17, and B5.13) using the paired-end reads (100 bp) and insert size of 170 bp in the Illumina Hiseq 2000 platform. After data filtering and quality control, reads were *de novo* assembled into contigs with MEGAHIT v1.0.4-beta (–kmin-1pass –presets meta-large –min-contigs-len 100) (Li et al., 2016) and only contigs longer than 500 bp were kept to do gene prediction with GeneMark.hmm version 2.7 days (-m MetaGeneMark_v1.mod) (Zhu et al., 2010). To construct a non-redundant gene set, predicted genes more than 100 bp were clustered with 95% identity and 90% alignment coverage by CD-HIT version 4.6 (cd-hit-para.pl –P cd-hit-est -n 8 –Q 30 –T SGE –S 3 -G 0 -aS 0.9 -c 0.95 -M 0 -T 0 -d 0 -r 1 -g 1) (Li and Godzik, 2006). Relative abundance profile of this gene set was obtained by following the method described by Qin et al. (2012). Taxonomic annotation was performed by aligning the amino acid sequences translated from genes to non-redundant protein database (nr database v20160509^3^) with BLAST (blastall 2.2.24, -e 1e-5 -b 50) and determined with LCA algorithm (first, filter with match percentage 80%, identity 65%, *e*-value within 10 times of the minimum *e*-value; then annotated to the least common taxonomic level). Functional annotation was performed by aligning the amino acid sequences in the KEGG database v81 using BLAST 2.2.31+ (blastp -outfmt 6 – *e*value 1e-5 -num_threads 6 num_alignments 50) and keeping the highest score hit with bit score greater than 60. We created a table with all information by combining the taxonomic and functional annotation results along with the abundance profile. After computing the proportion of all kingdoms, we kept the genes that were annotated to microbes, including bacteria, archaea, fungi, and viruses, for further functional analysis.

### Functional Analysis

As the starch and cellulose hydrolase activities (liquefaction, saccharification, and CA) are the key properties for starters quality, we focused on the relevant metabolic pathways. We categorized the microbial genes by their EC numbers and listed all the EC numbers involved in starch-glucose metabolism map according to the KEGG pathway and obtained the intersection. Thus, we got a specific starch-glucose map for the microbial community in our samples and analyzed the probable main pathway based on the relative abundance. In addition, the putative leading microbes in these metabolic processes were obtained by summarizing the taxonomic annotation. Similar analyses were performed for cellulose metabolism as well.

## Results

### The Microbial Profile of Immature and Mature Starters

**FIGURE 1:**
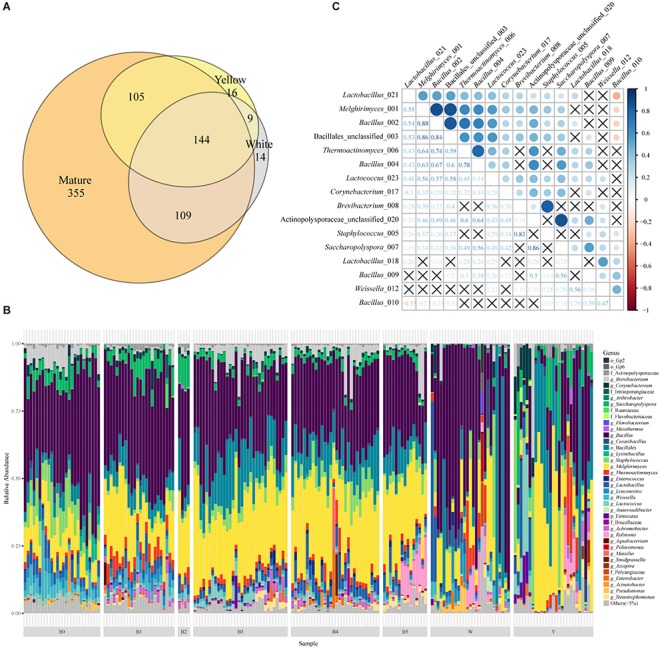
The taxonomic profile and important OTUs among Moutai starters (Daqu). **(A)** The Venn diagram of OTUs in yellow, white, and mature starters. **(B)** Taxonomic profile and the relative abundance of all 185 samples based on 16S rRNA metagenomic sequencing. Shown are the lowest taxonomic levels the OTUs can be annotated to, including phylum (p_), order (o_), family (f_), and genus (g_). “Others (<5%)” includes all taxa less than 5%. W and Y stand for white and yellow immature starters. B0 to B5 stands for six batches of mature starters. **(C)** The Spearman correlations between 16 core OTUs present in more than 95% mature starter samples (significance level is 0.05, *P* value is adjusted in the FDR method). The labels are composed of the taxa and the last three numbers of OTUs original names.

To illustrate the bacterial community composition of Moutai Daqu, we analyzed the 16S amplicon sequencing data of immature and mature starters and identified a total of 752 OTUs in 185 samples. Mature starters group contained the maximum number of OTUs (713), while white and yellow contained significantly lower 276 and 274 OTUs, respectively. Among them, 144 mutual OTUs shared by all three groups (Figure 1A) accounted for the majority (relative abundance >90%) in almost all samples (181/185 samples). The yellow and white group shared about 55% OTUs (153) which made up a considerable proportion (relative abundance >95% in 51 of 54 immature samples). Overall, the difference between yellow and white starters can be explained by two parts: divergent abundance of the shared half OTUs and uniqueness of the unshared half (pairwise comparisons of total OTUs with PERMANOVA and Mantel test, *P* value < 0.01; Wilcoxon rank sum test of single OTU, *P* value < 0.05, Supplementary Table S3). About 90% OTUs of immature starters were succeeded by mature starters. Interestingly, the mature starters contained 355 new OTUs absent in either immature group, which represented only a very small proportion (below 1% of the mature samples).

Next, we annotated OTUs with RDP version 16^4^ and discovered 19 phyla across all the samples. Actinobacteria, Firmicutes, and Proteobacteria were the main phyla. We found a similar pattern of bacterial composition among mature samples while high divergence in immature groups (Figure 1B). The dissimilarity between W and Y was obvious: the predominant genus of W was *Bacillus* while the majority of Y was *Melghirimyces*. Besides, the dissimilarity within each immature group was also noticeable from the poor clustering (Supplementary Figures S2A–C). In mature Daqu, the genera *Bacillus* and *Melghirimyces* and the order Bacillales (unclassified) were still the most abundant bacteria. Moreover, the microbial communities in mature starters were more consistent and had higher species richness than immature starters (Supplementary Figure S3; Tukey’s HSD test of Shannon index, most pairs’ *P* value < 0.01).

### The Core OTUs of Mature Starters

To discover the essential components of mature starters, we further explored the core microbiota (the OTUs present in more than 95% mature starter samples). Sixteen OTUs composed the mature core microbiota, four of which were annotated to Actinobacteria (all Actinomycetales) and twelve were Firmicutes (four Lactobacillales and eight Bacillales). With the exception of one OTU (*Bacillus*_010), all the 15 OTUs showed a significant positive correlation, especially for (1) among *Melghirimyces*_001, *Bacillus*_002, and Bacillales_unclassified_003, (2) between *Brevibacterium*_008 and *Staphylococcus*_005, and (3) between Acti- nopolysporaceae_unclassified_020 and *Saccharopolyspora*_007 (Figure 1C). These significant strong positive associations suggest a steady mutualistic relationship between these microbes in mature starters. In addition, three Bacillales OTUs (*Melghirimyces*_001, *Bacillus*_002, and *Bacillus*_004) were also present in at least 95% immature samples and the rest 13 OTUs were in at least half immature samples.

### Leave-One-Out Cross Validation of Immature Starters

**FIGURE 2:**
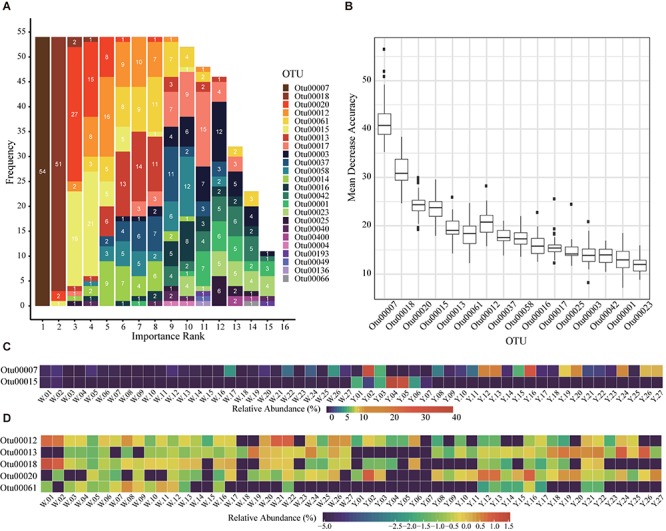
Seven marker OTUs selected with Leave-One-Out Cross Validation (LOOCV). **(A)** The rank of OTUs in LOOCV with random forest classifier. The labels are all feature OTUs in a total of 54 classifier. The *x* axis is the rank and the *y* axis is the frequency of each rank. The number in each bar is the frequency of each OTU in each rank. The OTUs with sum of numbers greater than 50 are identified as marker OTUs. **(B)** The mean decrease accuracy boxplot of OTUs in the 54 random forest classifiers for LOOCV. The present OTUs are used in more than 10 classifiers. **(C,D)** The relative abundance of the seven marker OTUs in 54 immature starters. **(C)** The relative abundances are percentages (x%). **(D)** The relative abundances are percentages transformed with formula log_10_ (relative abundance+1e-08).

Although microbes are considered as the core of starters, the empirical classification of immature starters is mainly based on their external feature: colors (Zhang, 2011). The permutational multivariate analysis of variance (PERMANOVA) and Mantel test showed that entire bacterial compositions were significantly different between white and yellow group (*p* < 0.01). Thus, we performed LOOCV on all 54 immature samples to select marker OTUs as microbial features of starters. We used 53 samples as a training set to do feature selection and build a random forest classifier; then we used the classifier to predict the left one sample and recorded the feature importance rank. After each sample had been tested, we found seven OTUs were selected as markers in more than 90% of all 54 rotations (Supplementary Table S4). Among them, OTU00007 classified as *Saccharopolyspora* was the most important feature every rotation and can be considered as the key distinguishing OTU between white and yellow starters (Figures 2A,B). In fact, its abundance was significantly higher in yellow starter group than white (Wilcoxon test, *p*-value < 0.001, Supplementary Table S3 and Figure 2C). As the whole microbial composition patterns of immature samples varied widely in either of two groups, it is reasonable that the division based on their maker OTUs heatmap was not apparent (Figures 2C,D). With the subtle differences in abundance between two groups considered overall, however, this 7-OTU combination became a more efficient classifying marker unit than any other combination or single OTU.

### Microbes Correlated With Physical and Chemical Properties of Mature Starters

**FIGURE 3:**
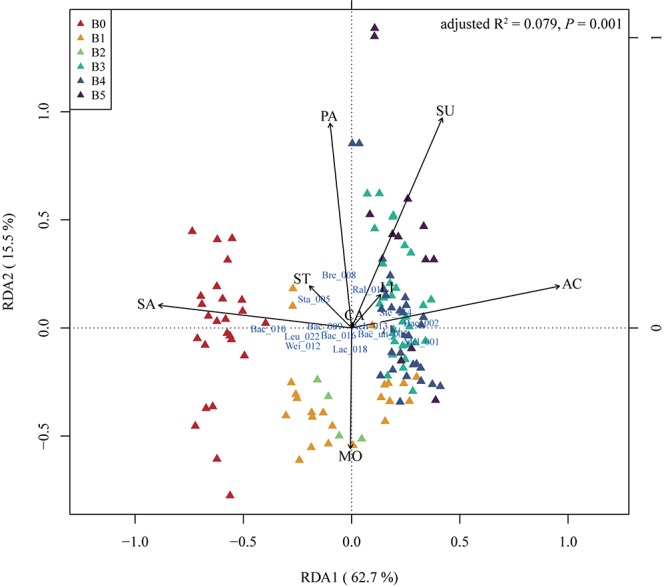
The redundancy analysis (RDA) triplot of the 126 mature samples. Hellinger-transformed OTUs table, constrained by eight physical and chemical properties, including acidity (AC), reducing sugar (SU), moisture (MO), starch (ST), saccharification (SA), liquefaction (LI), protease activity (PA), and cellulase activity (CA). The labels of OTUs are composed of the first three letters of taxa and the last three number of OTUs original names, “un” means unclassified. As there are too many OTUs to present, only OTUs with (weighted) orthonormal species scores of RDA1 and RDA2 greater than 0.15 are plotted. “Mel” – *Melghirimyces*, “Bac” – *Bacillus*, “Bac_un” – Bacillales_unclassified, “Sta” – *Staphylococcus*, “Bre” – *Brevibacterium*, “Ral” – *Ralstonia*, “Wei” – *Weissella*, “Ach” – *Achromobacter*, “Ste” – *Stenotrophomonas*, “Lac” – *Lactobacillus*, and “Leu” – *Leuconostoc*. B0 to B5 stands for six batches of mature starters.

Physical and chemical properties are the reference of traditional starter quality identification and related to microbial function in the starters. We measured eight indices including acidity (AC), reducing sugar fraction (SU), moisture (MO), starch fraction (ST), saccharification (SA), liquefaction (LI), PA, and CA of 126 mature starter samples (the data of the other five samples were missing) (methods in the Supplementary Material). Spearman’s correlation analysis showed no strong correlation between these properties but a weak positive relationship between acidity and sugar fraction, acidity and liquefaction, sugar fraction and starch fraction, at the 95% significance level after FDR (false discovery rate) adjustment (Supplementary Figure S4). In the RDA of the mature starters OTUs data constrained by physiochemical properties, B0 showed higher values along saccharification than other groups (*t*-test, *p*-value < 0.05, except B2), and almost all B1 and all B2 showed high moisture and low PA (Figure 3). B3, B4, and B5 showed high acidity and sugar fraction, low saccharification, opposite to B0. Furthermore, the Spearman’s rho was calculated between all OTUs and individual property, and the results showed only three properties (acidity, saccharification, and PA) had significant relationships with OTUs (Supplementary Table S2). The *Bacillus*_010, *Oceanobacillus*_034, and Bacillaceae_unclassified_038 showed a significant negative correlation with acidity, and positive correlation with saccharification, opposite to *Stenotrophomonas*_014.

### Microbial Gene Set Based on Metagenomic Sequencing

**FIGURE 4:**
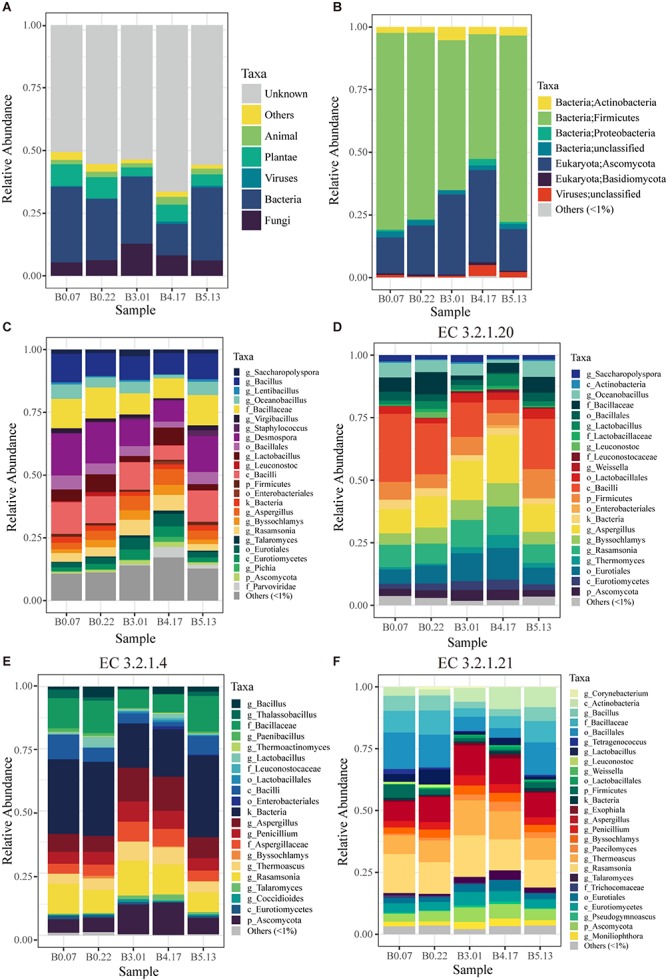
The taxonomic profiles of five mature samples based on metagenomic sequencing. **(A)** The profile of all data in kingdom level: “Unknown” means having no blast result in “nr” database; “Others” includes unclassified kingdom and protist. **(B)** The profile of microbial data (archaea, bacteria, fungi, and viruses) in phylum level: “unclassified” includes the data that have no annotation information in phylum level. **(C)** The profile of microbial data clustered by the lowest common level. **(D)** The microbial profile of alpha-glucosidase (EC 3.2.1.20) encoding genes grouped by the lowest annotation level. **(E,F)** The microbial profiles of enzyme encoding genes in cellulose hydrolysis, including endoglucanase (EC 3.2.1.4) and beta-glucosidase (EC 3.2.1.21). “Others (<1%)” includes all the taxonomic groups with relative abundance less than 1%.

The capabilities for compounds transformation in the fermented process is critical for the quality of liquor, which mainly attributed to the function of microorganisms. For fully characterizing the microbial functions in mature starters, we constructed a 1.1 million gene set based on metagenome data of 5 mature starters. In the original gene set, more than half of the genes had no annotation in the NCBI non-redundant protein (nr) database (version 20160509^5^), around 6%∼13% were from plant and animal, and 20%∼40% belonged to microbes (Figure 4A). Our analyses were based only on the microbial genes. The microbes of these five mature samples were from four domains (1.75e-05 ± 1.67e-05% Archaea, 74 ± 11% Bacteria, 25 ± 10% Fungi, and 1.71 ± 1.67% Viruses) composed of a total of 23 phyla (1 from archaea, 9 from fungi, and 13 from bacteria). The major phyla Ascomycota (Fungi) and Firmicutes (Bacteria) accounted for 87%∼94% microbes (Figure 4B). Representing around 96.6% of Fungi, the phylum Ascomycota in B3.01 (28.9%) or B4.17 (33.0%) was twice as great as in the rest three samples (B0.07 12.9%, B0.22 17.5%, and B5.13 14.6%), appearing a parabolic curve form: rising from B0 to B3, reaching a peak B4, and descending in B5. In Ascomycota phylum, *Aspergillus* (4.4 ± 1.5%), *Rasamsonia* (4.4 ± 1.7%), and *Byssochlamys* (2.8 ± 1.2%) were predominant genera and together accounted for more than half Ascomycota. Although there were 13 known phyla in Bacteria, about 91% of Bacteria belonged to the phylum Firmicutes. Moreover, the dominant orders of Firmicutes were Bacillales (48.0 ± 10.7%) and Lactobacillales (7.7 ± 3.2%). Overall, main bacterial genera were *Desmospora* (13.2 ± 3.7%), *Bacillus* (9.7 ± 1.1%), *Lactobacillus* (4.7 ± 2.3%), *Oceanobacillus* (4.2 ± 1.7%), *Saccharopolyspora* (1.7 ± 0.5%), and *Virgibacillus* (1.4 ± 0.5%). A total of 8840 KEGG (Kanehisa et al., 2017) functional orthologs (KO) were discovered among all five mature samples and 7885 KOs were shared, suggesting a consistency of fermentation function across different batches (Supplementary Figure S5).

### Enzymes and Microbes Profile of Starch Metabolism

**FIGURE 5:**
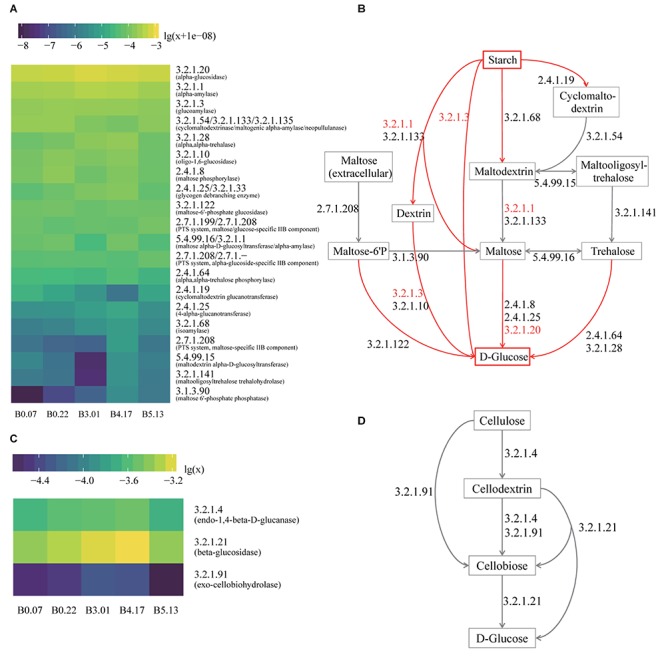
The functional analysis of five mature samples based on metagenome annotation. **(A)** The heatmap of all genes annotated to the enzymes in the hydrolysis of starch to glucose. The values are decimal abundances transformed with the formula log_10_(relative abundance+1e-08) (the sum of relative abundances of total genes including other enzymes equals to 1). The labels of *Y* axis are the EC numbers and enzyme names within parentheses. **(B)** The hydrolysis pathway of starch to glucose. The red arrows are the actual process of liquefaction and saccharification, which are the hydrolysis of the starch and the production of the glucose. The EC numbers in red refer to the top three enzymes with high gene abundance. **(C)** The heatmap of all genes annotated to the enzymes in cellulose hydrolysis. The values are decimal abundances transformed with the formula log_10_(relative abundance). **(D)** The hydrolysis pathway of cellulose to glucose, which is the process in CA detection.

Liquefaction and saccharification, in other words, starch hydrolysis and glucose production, are the essential processes in Moutai liquor fermentation. According to the KEGG database annotation, the most abundant enzyme-encoding genes in starch metabolism were found to be alpha-glucosidase (EC 3.2.1.20), alpha-amylase (EC 3.2.1.1), and glucoamylase (EC 3.2.1.3) genes in the five mature samples (Figures 5A,B). Alpha-glucosidase genes were three times as many as glucoamylase genes on average, suggesting the main pathway of the saccharification process in Moutai starters. The dominant microbes of alpha-glucosidase (3.2.1.20) genes were the class Bacilli, the genus *Aspergillus*, the genus *Rasamsonia*, and the order Eurotiales (Figure 4D). The high abundances of these microorganisms could be a clue to the high saccharifying power.

### Enzymes and Microbes Profile of Cellulose Metabolism

When raw materials are added in the Moutai liquor fermentation, three-quarters are usually the unground sorghum grains. Thus, cellulose hydrolysis is actually the first step in the fermentation process. Among the three cellulases annotated by KEGG database, beta-glucosidase (EC 3.2.1.21) genes were the most abundant, followed by endoglucanase (EC 3.2.1.4) genes (Figures 5C,D). The predominant microbes of beta-glucosidase genes were mainly fungi, such as the *Rasamsonia*, *Thermoascus*, and *Aspergillus* genera, taking 50∼70%, and the rest were bacteria classes Bacillales and Actinobacteria (Figure 4F). The taxa of endoglucanase genes were less than beta-glucosidase, mainly the unclassified Bacillaceae and the unclassified Bacteria, as well as several eukaryotes, including *Aspergillus*, *Rasamsonia*, and the unclassified Ascomycota (Figure 4E). These fungi and bacteria probably had important roles in cellulose degradation and are vital for fermentation.

## Discussion

Our present study offered a new insight into how the microbial diversity differed between the Moutai immature and mature starters, and identified the marker OTUs of immature starters as well as the core OTUs of mature starters, developing a primary numerical microbial standard for Moutai starters manufacture. To the best of our knowledge, this is the first study regarding the microbial composition of Moutai immature starters which fulfills the knowledge gap and provide vital information regarding the intermediate state of mature starter microbiota, which is important for liquor fermentation. Moreover, we explored the relationships between the microbes and physiochemical properties as well as the potential metabolic pathways. Employing both 16S rRNA sequencing and shotgun metagenome sequencing strategies, we exposed the unculturable and low-abundant part of the microbial community in Moutai starters, which was missed by previous studies with traditional techniques.

In immature starters, the shared half OTUs of white and yellow groups occupied the major proportion, while the total bacterial compositions and the abundance of several main OTUs were significantly different between two groups (PERMANOVA and Mantel test, *P* value < 0.01; Wilcoxon rank sum test, *P* value < 0.05, Supplementary Table S3). The similarities and differences of the two groups are reasonable as they are produced with the same raw materials and procedure, but in different positions and conditions: white starters were usually from the top layer of piles of starter bricks with lower temperature, lower moisture, and more air, while yellow starters were from the middle layer with the contrary conditions (Zhang, 2011).

The microbial community comparison between yellow and white starters is the new discovery of this study. The current color-based classification method of immature starters has been passed down through hundreds of generations. Moreover, according to experience, the high proportion (80%) of yellow starters is considered vital to both the quantity and quality of Moutai liquor production. Yet the relationships among the color, quality, and microbial community of immature starters are still unclear. The seven marker OTUs selected by LOOCV will be beneficial in identifying different types of immature starters and controlling their ratio. As the abundances of six markers OTUs were significantly different between yellow and white starters (Supplementary Table S3), further studies about fermentation ability divergence of immature starters can start with these microbes, promoting the establishment of immature starters quality standard. However, more immature samples are needed to verify these marker OTUs.

Interestingly, an OTU annotated to *Saccharopolyspora* was the most important feature during all the rotations in LOOCV, suggesting its potential commercial value. Earlier studies have demonstrated that *Saccharopolyspora* is the third largest of the nine dominant microbial clusters in a central black component of Moutai starter (Li et al., 2014). Colonizing the central part of Daqu brick, *Saccharopolyspora* was not considered relevant to the color of yellow starters directly, despite its higher abundance in yellow starters than in white starters. The Daqu color was attributed to the Maillard reaction, which will produce brown compounds when an amino acid and a reducing sugar are heated (Zheng et al., 2011). This reaction taking place in white starters (lower temperature and humidity) is too weak while in black starters (high temperature and humidity) is violent. Only when the Maillard reaction in the starters is moderate in the proper temperature and humidity, the Daqu is yellow and has strong soy sauce flavor. Besides, the genus *Saccharopolyspora*, including *S. hordei* and *S. rectivirgula* along with *Bacillus* has been reported to be the most abundant bacterial flora in wheat Qu as well (Guan et al., 2012). *Saccharopolyspora* sp. are known to produce a thermostable α-amylase enzyme which helps to hydrolyze starch to glucose, maltose, and maltotriose (Chakraborty et al., 2011). This finding implies that *Saccharopolyspora* is essential for generating flavoring substances in the downstream process of Moutai liquor production but further research is necessary.

In mature starters, six different batches had consistent bacterial diversity and shared 16 core OTUs. No significant differences were observed between most pairs of these six batches with Tukey’s HSD (honestly significant difference) test (Shannon index, *P* value < 0.01, except B0–B3, B0–B4, B1–B3, and B1–B4), although the result of overall ANOVA (analysis of variance) showed differences existed. A similar conclusion can be drawn by PCoA based on the css-transformed OTUs abundances (Supplementary Figures S2D–F). The differences between the first two batches and the rest four batches may be explained by the different temperature or humidity of months when they were produced.

Overall, the high-abundant OTUs of mature starters were mainly inherited from immature starters, while their abundances changed in the 6 months of storage, and some low abundant OTUs unique to mature starters were considered to come from the surrounding environment during this half year. Although yellow starters occupied 80% in immature starters, mature starters, as the mixture of immature starters, obtained a more balanced microbial community instead of being the same as yellow starters. Thus, we assume the half-year storage is an essential stage allowing the microbial communities in starters attract members from the background and gradually reach a balance. Previous studies have also indicated that the special microbial community constrained by the particular natural environment (climate, water, etc.) of Moutai Town, Guizhou Province, makes the liquor unreproducible in anywhere else, even if using the same raw materials and process(Wang et al., 2008, 2017, 2018). But this conjecture needs to be confirmed in future studies.

Based on 16S amplicon analysis, *Bacillus*, *Melghirimyces*, and the unclassified Bacillales were found to be the most abundant taxa in mature starters. A similar dominance of *Bacillus* has also been observed in other types of starters (Rice wine, Fen liquor) (Zheng et al., 2015; Cai et al., 2018). The strong hydrolytic capabilities of *Bacillus* species are fundamental to the following formation of flavor compounds (Beaumont, 2002), while their abilities to survive in high-temperature condition contribute to their dominance (Wang et al., 2008). For metagenomics analysis, we selected five mature samples, including two B0 samples (B0.07 and B0.22), one B3 (B3.01), one B4 (B4.17), and one B5 (B5.13). These samples had no significant sensory difference with the rest of mature starters. We selected them because these four batches are quite important for Moutai liquor production. If added in the first cycle, the B0 starters can create an essential microbial and enzymatic basis for the entire liquor fermentation. Moreover, the B3–B5 displayed the highest output cycles among all. Liquor batches steamed from these three cycles are the main components of final Moutai liquor, not only because they take up the largest proportion (respectively, 25, 22, and 15%), but also because their flavors match perfectly with the standard of Moutai liquor (Guizhou Provincial Bureau of Quality and Technical Supervision, 2018). Therefore, it is important to study the microbial communities of starter batches added in these cycles. From the composition result (Figure 4C), we found the main structures of five samples were similar: the majority of microbes were bacteria and minority were fungi, and the dominant phyla were Firmicutes and Ascomycota. The main microbes, such as *Desmospora*, *Bacillus*, *Aspergillus*, and *Rasamsonia*, were mutual among all the five samples. The similarity is reasonable as they were produced by the same process in a relatively enclosed environment. However, the proportion of Fungi in B3.01 or B4.17 was almost twice as great as in the rest three samples, respectively. We attributed the discrepancy to different weather conditions, such as temperature and humidity, of the months they were produced. B0 starters were produced in the winter, while B3–B5 were produced in April-June. According to the weather history records of Guiyang (capital city of Guizhou) from 1961–1990^6^, the average temperatures of January, April, May, and June were 4.8°C, 16.4°C, 21.1°C, and 24.5°C; the monthly precipitation records were 39.0 mm, 164.4 mm, 207.9 mm, and 196.0 mm. We speculate that the rising temperatures and rainfalls from January to May might have produced a more favorable environment for Fungi. However, this could not explain the decrease from B4 to B5. We were also unable to acquire the weather records of actual production months in Moutai town, which may slightly differ from the records we obtained. While some *Saccharomyces* are pivotal and strongly linked to specific wine types in the traditional wine industry, non-*Saccharomyces* yeasts such as *Aspergillus* were dominant molds in both wine and Daqu (Pretorius, 2000; Chen and Zhu, 2013). A similar pattern can be observed in our study, where *Aspergillus*, *Rasamsonia*, and other Eurotiomycetes are the predominant fungi (Figure 4C).

Physical and chemical properties importantly influence and are influenced by the microbial community in starters (Cai et al., 2018). Here we explored the correlation of 126 mature starter cultures samples with eight physiochemical indices. With RDA and Spearman correlation analysis, we found that different batches of mature samples had their special features in some physiochemical properties, and several OTUs had significant relationships with the properties. According to the RDA results and Student’s *t*-test, B0 showed significantly stronger saccharifying power than any other group (*p*-value < 0.05, except B2). Meanwhile, saccharifying power had a significant positive correlation with OTU00010 (*Bacillus*) based on Spearman’s rho (Supplementary Table S2), and the relative abundances of OTU00010 (*Bacillus*) in B0 were significantly higher than the rest five batches (Wilcoxon test, *p*-value < 0.01). *Bacillus* are well known for its biotechnological application and is widely utilized as the saccharifying biological agent (Beaumont, 2002; Patel et al., 2005; Kapoor et al., 2008). In our analysis, the OTU00010 (*Bacillus*) also contributed to the stronger saccharifying power of B0, while the biochemical process behind this remains to be elucidated in subsequent experiments. As OTU00010 (*Bacillus*) was the only core OTU negatively correlated with other core OTUs (Figure 1C), the potential inhibition of other core OTUs on OTU00010 (*Bacillus*) probably helps to regulate the saccharification in mature starters. These results can also be applied to minimize the difference between different batches. Besides, alpha-glucosidase (EC 3.2.1.20) was found to be the most abundant enzyme in the starch-glucose pathway in all mature samples, which provide a potential starting point for solving the problem with abnormal saccharifying power of mature starters.

Daqu is one of the most important factors influencing Moutai liquor flavor. There is a saying goes, “Daqu is the bone structure of liquor”. On the one hand, Moutai Daqu powder makes up half the percentage of Moutai liquor’s total material. On the other hand, when Daqu powder is mixed with sorghum grains, the microbes and enzymes also join in and create the fermentation environment for following production process. Our study revealed that the genus *Bacillus* and other genera under Bacillales were dominant (44.8 ± 10.1%) in the mature starters, while all fungi accounted for about a quintile (21.9 ± 9.0%); the abundance of *Bacillus* (9.7 ± 1.1%) was about twice as great as *Lactobacillus* (4.7 ± 2.3%) and *Aspergillus* (4.4 ± 1.5%). Since the mature starters are very dry (moisture <10%) and fermented grains are moist, it is reasonable to assume fungi will possibly take over bacteria and become the dominant microbes when starters are just mixed with fermented grains and the moisture and temperature conditions become more suitable. Afterward, there is a dynamic microbial composition during a cycle of the fermentation process. The previous study indicated the core functional microbe changed from *Schizosaccharomyces* to Lactobacillus within a 30-day fermentation round, during which the flavor compound shifted from alcohol to acid (Song et al., 2017). However, the authors did not elucidate which cycle of pit fermentation they sampled. Actually, a different cycle of fermentation may have different volatiles composition due to the gradual degradation of starch. Besides, the conclusion of core microbial conversion was drawn indirectly from the correlation analysis of amplicon sequencing data and metatranscriptomic data, which is not as solid as the direct metagenomic data evidence.

Despite the microbial succession, analyzing starter microbiota – the starting point of fermentation – is still critical to studies of final liquor flavor. *Bacillus licheniformis*, isolated from Moutai mature starter, produced an abundance of volatile C4 compounds, mainly including pyrazines, volatile acids, aromatic and phenolic compounds, in the fermentation process and led to a liquor getting higher evaluation than the control without inoculating it (Zhang et al., 2013). In a study of fortified strong-flavor Daqu inoculated with two *Bacillus* species, *Lactobacillus* and *Candida* were reported to have positive relationships with most esters in the bottom layer of fermented grains; *Bacillus* and *Aspergillus* had strong positive relationships with aromatic compounds, such as benzene-ethanol and ethyl phenylacetate, which contribute to smells of rose and honey, in the middle of fermented grains as well as the correlative liquor (He et al., 2019). With only 16S and ITS data, however, this study could not compare the importance of bacteria with fungi in the contribution of flavor compounds. The authors also did not explain the possible reasons that 100% fortified Daqu led to a decrease of volatiles than 50% fortified Daqu instead of increase. In our study, a core OTU of *Bacillus* (OTU00010) was found to be negatively correlated with other core OTUs of *Bacillus* in mature starters. This contradiction inspired us to explore the role of every single species meticulously and try to figure out their interaction, instead of considering a genus roughly as a whole.

## Conclusion

Moutai liquor manufacture relies on traditional experiences. While the traditional method keeps the classic flavor of the liquor, it lacks full comprehension of the microbial communities and activities during the entire fermentation process, which are essential to facilitate liquor quality control. This study revealed the microbial profiles of the Moutai starters in different periods and analyzed the relationship between microbes and enzymes critical to fermentation. The unique microbial features of immature and mature starters are anticipated to contribute to a primary standard for different types of starters, helping maintain the stability of Moutai liquor quantity and flavor.

## Data Availability

The datasets generated for this study can be accessed from the CNGB Nucleotide Sequence Archive (https://db.cngb.org/cnsa/) under the Project accession number CNP0000316. They can also be accessed from the European Nucleotide Archive online repository (https://www.ebi.ac.uk/ena) under the Project accession number PRJEB30956.

## Author Contributions

FY, Y-YW, TJ, and HL established the concept and analyzed the framework of the study. FY, R-YL, TJ, and H-YW collected the samples. JX (11th author) and JX (12th author) generated the data, including DNA extraction, library construction, and sequencing. S-HG, Y-YW, S-LL, and P-FZ performed the bioinformatics analysis. S-HG, Y-YW, SS, HL, FY, LW, and XL wrote and edited the manuscript.

## Conflict of Interest Statement

FY, R-YL, H-YW, and LW are employed by company China Kweichow Moutai Distillery Co., Ltd. The remaining authors declare that the research was conducted in the absence of any commercial or financial relationships that could be construed as a potential conflict of interest.

## Footnotes

1 https://www.mothur.org/wiki/454_SOP

2 https://CRAN.R-project.org/package=vegan

3 ftp://ftp.ncbi.nlm.nih.gov/blast/db/

4 https://mothur.org/w/images/d/dc/Trainset16_022016.rdp.tgz

5 ftp://ftp.ncbi.nlm.nih.gov/blast/db/

6 https://www.hko.gov.hk/wxinfo/climat/world/chi/asia/china/guiyang_c.htm

## References

[B1] Beaumont M. (2002). Flavouring composition prepared by fermentation with *Bacillus* spp. *Int. J. Food Microbiol.* 75 189–196. 10.1016/s0168-1605(01)00706-1 12036142

[B2] Beller H. R. Han R. Karaoz U. Lim H. Brodie E. L. (2012). Genomic and physiological characterization of the chromate-reducing, aquifer-derived firmicute *Pelosinus* sp. strain HCF1. *Appl. Environ. Microbiol.* 79 63–73. 10.1128/AEM.02496-12 23064329 PMC3536105

[B3] Cai H. Zhang T. Zhang Q. Luo J. Cai C. Mao J. (2018). Microbial diversity and chemical analysis of the starters used in traditional Chinese sweet rice wine. *Food Microbiol.* 73 319–326. 10.1016/j.fm.2018.02.002 29526219

[B4] Caporaso J. G. Kuczynski J. Stombaugh J. Bittinger K. Bushman F. D. Costello E. K. (2010). QIIME allows analysis of high-throughput community sequencing data. *Nat. Methods* 7 335–336. 20383131 10.1038/nmeth.f.303 PMC3156573

[B5] Caporaso J. G. Lauber C. L. Walters W. A. Berg-Lyons D. Lozupone C. A. Turnbaugh P. J. (2011). Global patterns of 16S rRNA diversity at a depth of millions of sequences per sample. *Proc. Natl. Acad. Sci. U.S.A.* 108(Suppl. 1), 4516–4522. 10.1073/pnas.1000080107 20534432 PMC3063599

[B6] Chakraborty S. Khopade A. Biao R. Jian W. Liu X.-Y. Mahadik K. (2011). Characterization and stability studies on surfactant, detergent and oxidant stable α-amylase from marine haloalkaliphilic *Saccharopolyspora* sp. A9. *J. Mol. Catal. B Enzym.* 68 52–58. 10.1016/j.molcatb.2010.09.009

[B7] Chen J. Zhu Y. (2013). *Solid State Fermentation for Foods and Beverages.* Boca Raton, FL: CRC Press.

[B8] Guan Z. B. Zhang Z. H. Cao Y. Chen L. L. Xie G. F. Lu J. (2012). Analysis and comparison of bacterial communities in two types of ‘wheat Qu’, the starter culture of Shaoxing rice wine, using nested PCR-DGGE. *J. Inst. Brew.* 118 127–132. 10.1002/jib.4

[B9] Guizhou Provincial Bureau of Quality and Technical Supervision (2018). *Technology Specification for Production of Jiang Xiang Xing Baijiu of Daqu (DB52/T 873-2018).* Guizhou: Guizhou Provincial Bureau of Quality and Technical Supervision.

[B10] Guo M.-Y. Huo D.-Q. Ghai R. Rodriguez-Valera F. Shen C.-H. Zhang N. (2014). Metagenomics of ancient fermentation pits used for the production of Chinese strong-aroma liquor. *Genome Announc.* 2:e1045-14. 10.1128/genomeA.01045-14 25342677 PMC4208321

[B11] He G. Huang J. Zhou R. Wu C. Jin Y. (2019). Effect of fortified daqu on the microbial community and flavor in chinese strong-flavor liquor brewing process. *Front. Microbiol.* 10:56. 10.3389/fmicb.2019.00056 30761106 PMC6361764

[B12] Hong X. Chen J. Liu L. Wu H. Tan H. Xie G. (2016). Metagenomic sequencing reveals the relationship between microbiota composition and quality of Chinese Rice Wine. *Sci. Rep.* 6:26621. 10.1038/srep26621 27241862 PMC4886530

[B13] Huang Y. Yi Z. Jin Y. Zhao Y. He K. Liu D. (2017). New microbial resource: microbial diversity, function and dynamics in Chinese liquor starter. *Sci. Rep.* 7:14577. 10.1038/s41598-017-14968-8 29109406 PMC5674051

[B14] Kanehisa M. Furumichi M. Tanabe M. Sato Y. Morishima K. (2017). KEGG: new perspectives on genomes, pathways, diseases and drugs. *Nucleic Acids Res.* 45 D353–D361. 10.1093/nar/gkw1092 27899662 PMC5210567

[B15] Kapoor M. Nair L. M. Kuhad R. C. (2008). Cost-effective xylanase production from free and immobilized *Bacillus pumilus* strain MK001 and its application in saccharification of *Prosopis juliflora*. *Biochem. Eng. J.* 38 88–97. 10.1016/j.bej.2007.06.009

[B16] Li D. Luo R. Liu C. M. Leung C. M. Ting H. F. Sadakane K. (2016). MEGAHIT v1.0: a fast and scalable metagenome assembler driven by advanced methodologies and community practices. *Methods* 102 3–11. 10.1016/j.ymeth.2016.02.020 27012178

[B17] Li H. Lian B. Ding Y. Nie C. Zhang Q. (2014). Bacterial diversity in the central black component of maotai daqu and its flavor analysis. *Ann. Microbiol.* 64 1659–1669. 10.1007/s13213-014-0809-z

[B18] Li W. Godzik A. (2006). Cd-hit: a fast program for clustering and comparing large sets of protein or nucleotide sequences. *Bioinformatics* 22 1658–1659. 10.1093/bioinformatics/btl158 16731699

[B19] Li X.-R. Ma E.-B. Yan L.-Z. Meng H. Du X.-W. Quan Z.-X. (2013). Bacterial and fungal diversity in the starter production process of Fen liquor, a traditional Chinese liquor. *J. Microbiol.* 51 430–438. 10.1007/s12275-013-2640-9 23990293

[B20] Liaw A. Wiener M. (2002). Classification and regression by random forest. *R News* 2 18–22.

[B21] Lv X.-C. Weng X. Zhang W. Rao P.-F. Ni L. (2012). Microbial diversity of traditional fermentation starters for Hong Qu glutinous rice wine as determined by PCR-mediated DGGE. *Food Control* 28 426–434. 10.1016/j.foodcont.2012.05.025

[B22] Miao Y. (2013). “Climate and Maotai Wine,” in *Meteorological Knowledge (Qixiang Zhishi)*, ed. Chen Y.-F. (Beijing: China Meteorological Administration).

[B23] Patel M. A. Ou M. S. Ingram L. O. Shanmugam K. (2005). Simultaneous saccharification and co–fermentation of crystalline cellulose and sugar cane bagasse hemicellulose hydrolysate to lactate by a thermotolerant acidophilic *Bacillus* sp. *Biotechnol. Prog.* 21 1453–1460. 10.1021/bp0400339 16209550

[B24] Paulson J. N. Stine O. C. Bravo H. C. Pop M. (2013). Differential abundance analysis for microbial marker-gene surveys. *Nat. Methods* 10 1200–1202. 10.1038/nmeth.2658 24076764 PMC4010126

[B25] Pretorius I. S. (2000). Tailoring wine yeast for the new millennium: novel approaches to the ancient art of winemaking. *Yeast* 16 675–729. 10.1002/1097-0061(20000615)16:8<675::aid-yea585>3.3.co;2-2 10861899

[B26] Qin J. Li Y. Cai Z. Li S. Zhu J. Zhang F. (2012). A metagenome-wide association study of gut microbiota in type 2 diabetes. *Nature* 490:55. 10.1038/nature11450 23023125

[B27] Schloss P. D. Gevers D. Westcott S. L. (2011). Reducing the effects of PCR amplification and sequencing artifacts on 16S rRNA-based studies. *PLoS One* 6:e27310. 10.1371/journal.pone.0027310 22194782 PMC3237409

[B28] Schloss P. D. Westcott S. L. Ryabin T. Hall J. R. Hartmann M. Hollister E. B. (2009). Introducing mothur: open-source, platform-independent, community-supported software for describing and comparing microbial communities. *Appl. Environ. Microbiol.* 75 7537–7541. 10.1128/AEM.01541-09 19801464 PMC2786419

[B29] Song Z. Du H. Zhang Y. Xu Y. (2017). Unraveling core functional microbiota in traditional solid-state fermentation by high-throughput amplicons and metatranscriptomics sequencing. *Front. Microbiol.* 8:1294. 10.3389/fmicb.2017.01294 28769888 PMC5509801

[B30] Tao Y. Li J. Rui J. Xu Z. Zhou Y. Hu X. (2014). Prokaryotic communities in pit mud from different-aged cellars used for the production of Chinese strong-flavor liquor. *Appl. Environ. Microbiol.* 80 2254–2260. 10.1128/aem.04070-13 24487528 PMC3993157

[B31] Wang C.-L. Shi D.-J. Gong G.-L. (2008). Microorganisms in daqu: a starter culture of Chinese maotai-flavor liquor. *World J. Microbiol. Biotechnol.* 24 2183–2190. 10.1007/s10295-009-0661-5 19904566

[B32] Wang D. Chen L. Yang F. Wang H. Wang L. (2019). Yeasts and their importance to the flavour of traditional Chinese liquor: a review. *J. Inst. Brew.* 125 214–221. 10.1002/jib.552

[B33] Wang L. Wang Y. Y. Wang D. Q. Xu J. Yang F. Liu G. (2015). Dynamic changes in the bacterial community in moutai liquor fermentation process characterized by deep sequencing. *J. Inst. Brew.* 121 603–608. 10.1002/jib.259

[B34] Wang Q. Zhang H. Liu X. (2016). Microbial community composition associated with Maotai liquor fermentation. *J. food Sci.* 81 M1485–M1494. 10.1111/1750-3841.13319 27122124

[B35] Wang X. Ban S. Hu B. Qiu S. Zhou H. (2017). Bacterial diversity of moutai–flavour daqu based on high-throughput sequencing method. *J. Inst. Brew.* 123 138–143. 10.1002/jib.391

[B36] Wang X. D. Ban S. D. Qiu S. Y. (2018). Analysis of the mould microbiome and exogenous enzyme production in moutai-flavor daqu. *J. Inst. Brew.* 124 91–99. 10.1002/jib.467

[B37] Xie G. Wang L. Gao Q. Yu W. Hong X. Zhao L. (2013). Microbial community structure in fermentation process of shaoxing rice wine by illumina-based metagenomic sequencing. *J. Sci. Food Agric.* 93 3121–3125. 10.1002/jsfa.6058 23553745

[B38] Zhang L. (2011). *Fermented Food Technology.* Beijing: China Light Industry Press.

[B39] Zhang R. Wu Q. Xu Y. (2013). Aroma characteristics of moutai-flavour liquor produced with *Bacillus licheniformis* by solid-state fermentation. *Lett. Appl. Microbiol.* 57 11–18. 10.1111/lam.12087 23594087

[B40] Zhang X. Zhao J. Du X. (2014). Barcoded pyrosequencing analysis of the bacterial community of daqu for light-flavour Chinese liquor. *Lett. Appl. Microbiol.* 58 549–555. 10.1111/lam.12225 24471485

[B41] Zheng X.-W. Han B.-Z. (2016). Baijiu, Chinese liquor: history, classification and manufacture. *J. Ethn. Foods* 3 19–25. 10.1016/j.jef.2016.03.001

[B42] Zheng X. W. Tabrizi M. R. Nout M. R. Han B. Z. (2011). Daqu–a traditional Chinese liquor fermentation starter. *J. Inst. Brew.* 117 82–90. 10.1002/j.2050-0416.2011.tb00447.x

[B43] Zheng X.-W. Yan Z. Nout M. R. Boekhout T. Han B.-Z. Zwietering M. H. (2015). Characterization of the microbial community in different types of daqu samples as revealed by 16S rRNA and 26S rRNA gene clone libraries. *World J. Microbiol. Biotechnol.* 31 199–208. 10.1007/s11274-014-1776-z 25395233

[B44] Zhu W. Lomsadze A. Borodovsky M. (2010). Ab initio gene identification in metagenomic sequences. *Nucleic Acids Res.* 38:e132. 10.1093/nar/gkq275 20403810 PMC2896542

