# Supplementary figures and images for: Deciphering the Composition and Functional Profile of the Microbial Communities in Chinese Moutai Liquor Starters
Source: Front Microbiol. 2019 Jul 4;10:1540. doi: 10.3389/fmicb.2019.01540 (PMC6620787; doi:10.3389/fmicb.2019.01540)

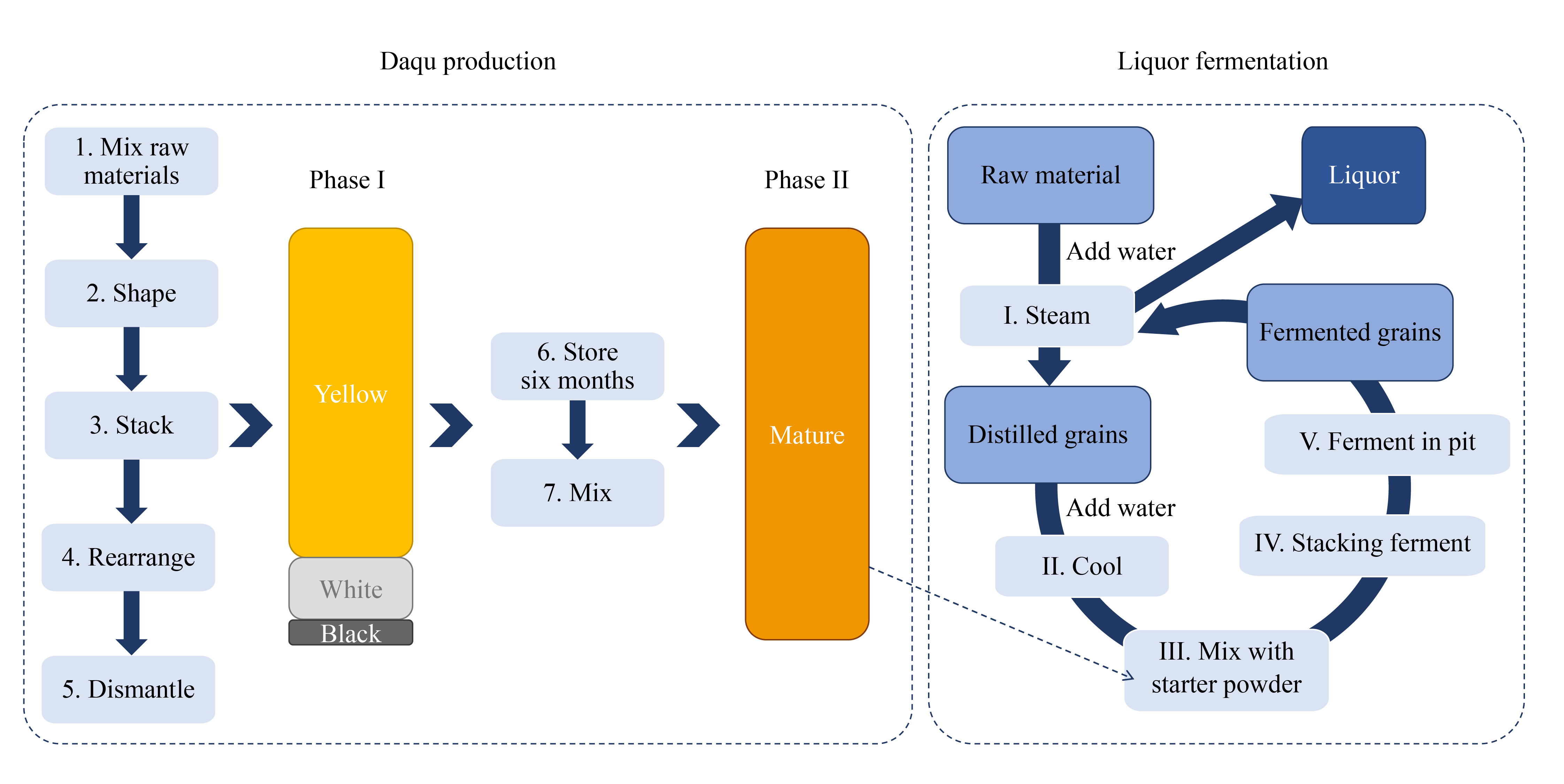

Supplement: Supplementary file 1 [file Image_1.JPEG]

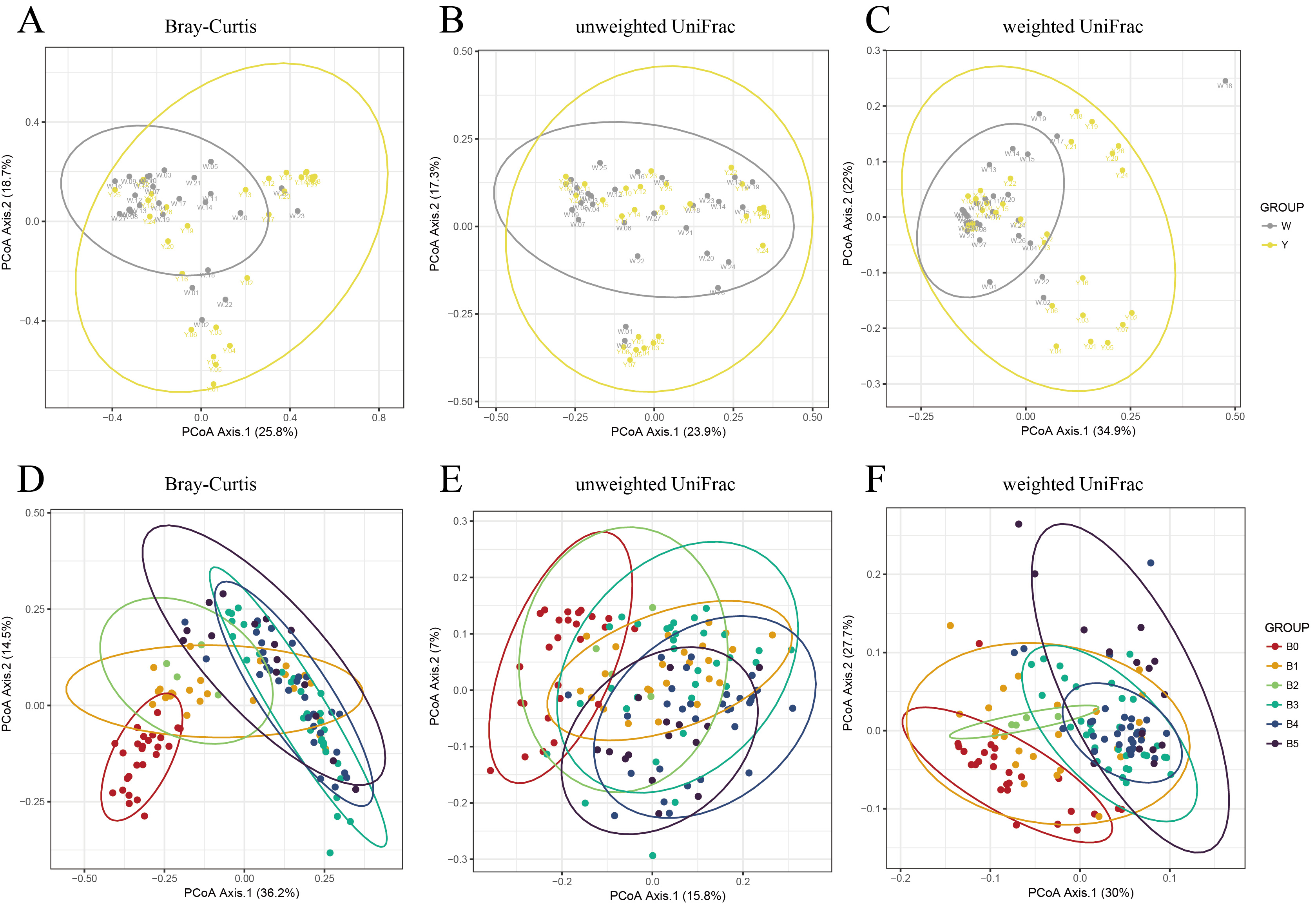

Supplement: Supplementary file 2 [file Image_2.JPEG]

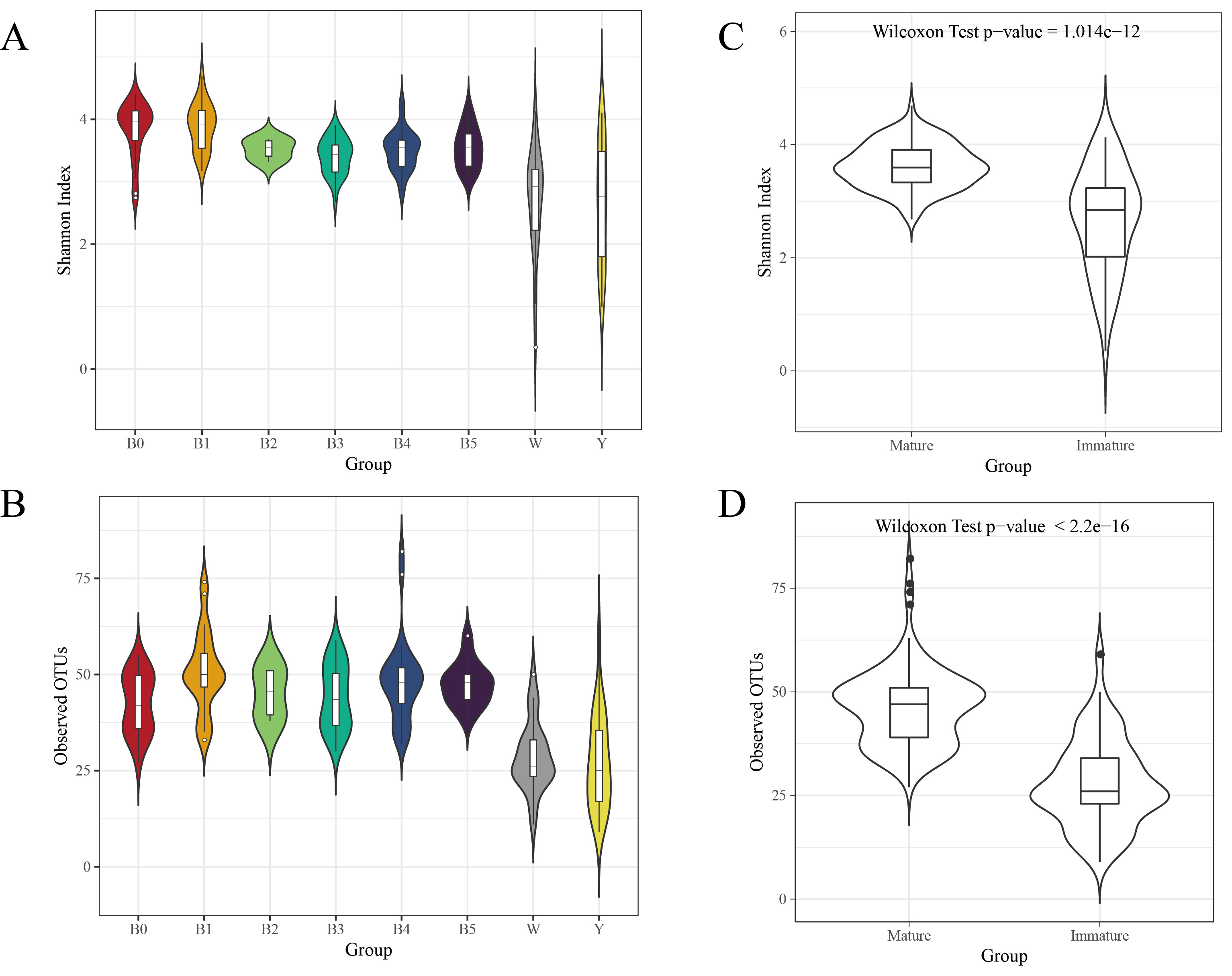

Supplement: Supplementary file 3 [file Image_3.JPEG]

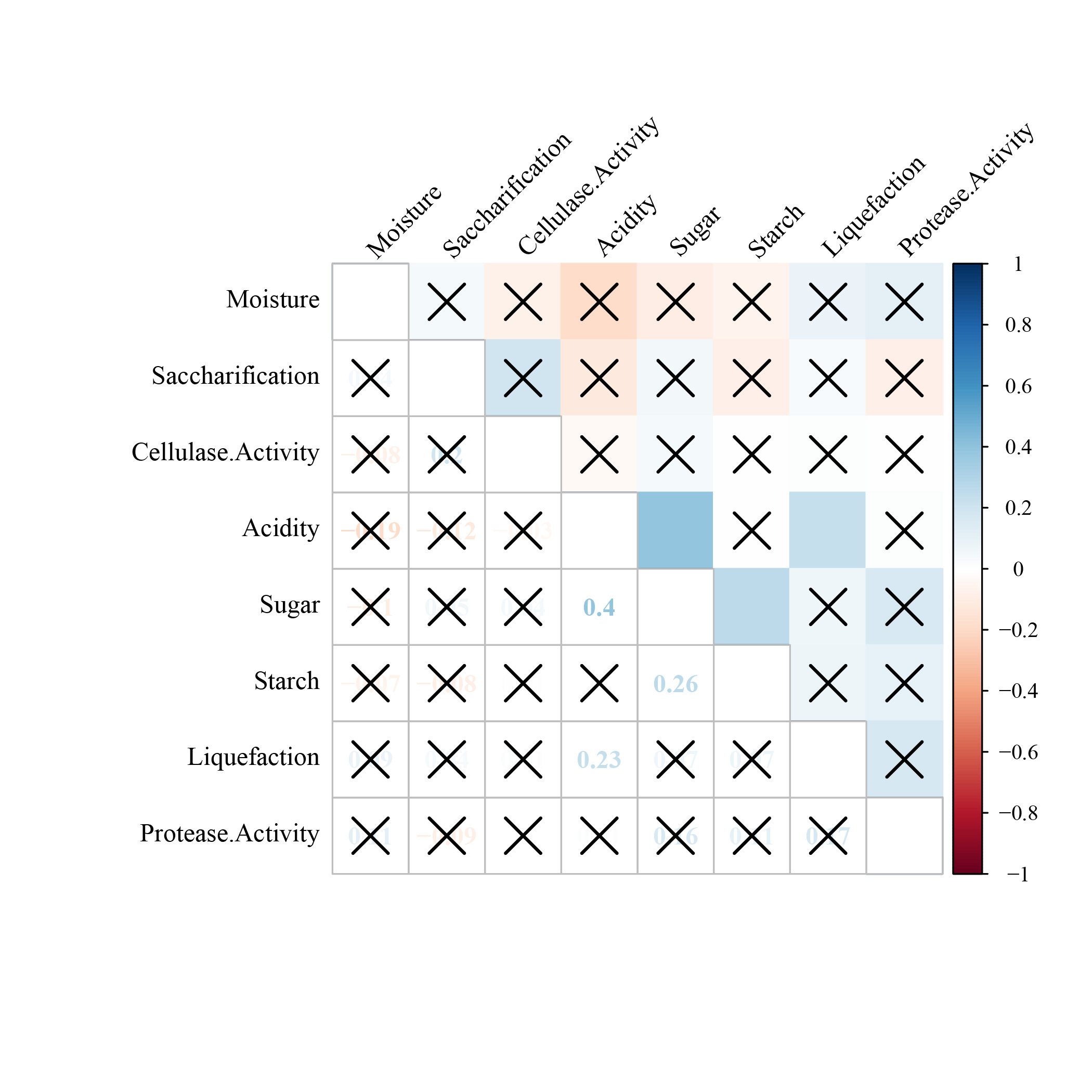

Supplement: Supplementary file 4 [file Image_4.JPEG]

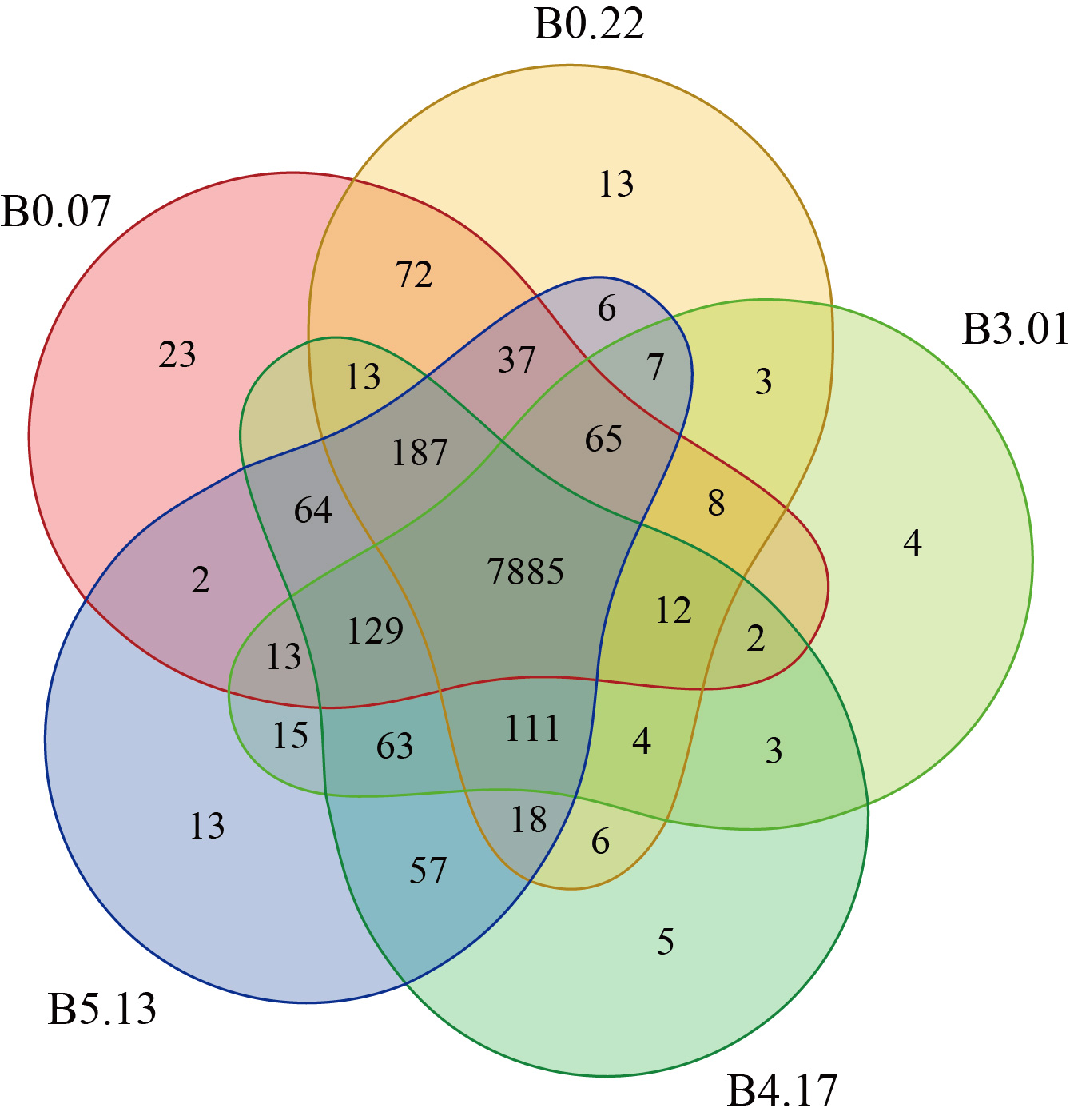

Supplement: Supplementary file 5 [file Image_5.JPEG]
