# Supplementary material for: Deciphering the Composition and Functional Profile of the Microbial Communities in Chinese Moutai Liquor Starters
Source: Front Microbiol. 2019 Jul 4;10:1540. doi: 10.3389/fmicb.2019.01540 (PMC6620787; doi:10.3389/fmicb.2019.01540)
Supplement: Supplementary file 6 [file Data_Sheet_1.pdf]

Supplementary Material

1 Measure Methods of Eight Physical and Chemical Properties

The methods (a-f) follow the Industry Standard (Ministry of Industry and Information Technology of China, 2011):

(a) Acidity: 10 g starter was mixed with 100 mL water, filtered with two-layer gauze, titrated with 0.1 mol/L NaOH, and then the acidity was calculated based on the used volume of NaOH solution;

(b) Reducing sugar: 10 g starter was mixed with 100 mL water, filtered with two-layer gauze, titrated with Fehling's solution, and the mass of reducing sugar was calculated based on the used volume of Fehling's solution;

(c) Moisture: 10 g of the starter was heat dried to a constant weight and the lost weight was calculated;

(d) Starch fraction: weight 10 g starter, remove lipid with methanol solution and remove soluble sugar with ethanol solution, flush the remains with 50 mL water, keep the liquid in the boiling water bath for gelatinization, cool down to 60°C, add α-amylase solution and hydrolyze the starch in the thermostat water bath (55 ℃ ~ 60 ℃), add a drop of iodine solution, keep doing the gelatinization-hydrolysis process until the hydrolysate dose not turn to blue when adding the iodine solution, boil the final hydrolysate, cool down and transfer to a volumetric flask, mix up and filter, add hydrochloric acid solution to the filtered liquid, reflux 1h in the boiling water bath with reflux condensing tube, cool down and add two drops of methylene blue indicator solution, neutralize with the NaOH solution, transfer the solution to a 1000 mL volumetric flask and dilute to volume, titrate with Fehling's solution and calculate the mass of starch with the used volume of Fehling's solution;

(e) Saccharification: 10 g starter was mixed with 85 mL water and 10 mL acetic acid - sodium acetate buffer solution, and kept in a 35 ℃ thermostat water bath for 1h and filtered. The 5 mL filtrate was then mixed with 20 mL 2% soluble starch solution and 5 mL water, and again kept in a 35 ℃ thermostat water bath for 1h. The resulting solution was mixed with Fehling's solution, and titrated with 0.2% standard glucose solution until the blue color disappear. The saccharifying power was calculated with respect to the used volume of the glucose solution;

(f) Liquefaction: weight 10 g starter and mix with 35 ℃ water, add 20 mL pH 6.0 citric acid - sodium hydrogen phosphate buffer, soak for 1h (mix every 15min), filter and get the enzyme solution, mix 20 mL 2% soluble starch solution and 5 mL pH 6.0 citric acid - sodium hydrogen phosphate buffer and heat 10min in a 60 ℃ water bath, add accurate 5 mL enzyme solution and begin timing, totally mix and take about 0.5 mL reaction mixture to a blank hole with 1.5 mL iodine solution on a white porcelain plate, stop timing when the solution color is from blue to red and finally the same as standard colorimetric solution color, calculate the liquefying power with the consumed time;

(g) Protease activity: use the method in the standard (Shandong Bureau of Quality and Technical Supervision, 2009), defined as the mass (ug) of tyrosine hydrolyzed from casein per minute by 1 g starter at 40 ℃ pH 7.2;

(h) Cellulase activity: measured with improved Lane-Eynon titration method using carboxymethyl cellulose as substrate, defined as the mass (mg) of glucose hydrolyzed from cellulose sodium per minute by 1 g starter at 45 ℃ pH 4.6 (Standardization Administration of the People's Republic of China (SAC), 2008).

2 Supplementary Figures

Figure S1 Manufacturing processes of Moutai starters (Daqu) and liquor. In phase I, the starters are called immature starters, which are just made by one-month fermentation of wheat grains. They can be classified into three groups by their color: yellow starters (80~85%), white starters (10~15%), and black starters (<1%). Yellow starters are properly fermented and have strong soy sauce flavor, while white starters are under-fermented and black are over-fermented. We sampled 27 yellow starters and 27 white starters. In phase II, the starters are the powder mixture of all immature starters that have been stored for six months, called mature starters. 'Mature' means the starters are ready to ferment. As the Moutai liquor fermentation process is composed of repeated monthlong cycles (batches), a batch of mature starters freshly produced each month will be added in a cycle just in time. We sampled mature starters from six batches and named them B0~B5.

Figure S2 The PCoA plots of Moutai starters based on relative abundance, (A-C) of immature starters and (D-F) of six batches of mature starters. Bray-Curtis distance (A and D); unweighted UniFrac distance (B and E); weighted UniFrac distance (C and F). Mature starters: B0-B5; immature starters: W, Y.

Figure S3 The spearman's correlation of eight physicochemical properties. The blue color means positive correlation while red means negative correlation. The X mark means the FDR-adjusted *P* value of this Spearman's rho is greater than 0.05.

Figure S4 The Venn diagram of KOs in five mature starters. Both B0.07 and B0.22 were the samples of batch 0, B3.01 of batch 3, B4.17 of batch 4, B5.13 of batch 5.

Figure S5 The comparison of alpha diversity indices between mature and immature starters. Shannon index (A) and observed OTUs numbers (B) of 8 groups of starters with each batch of mature starters as well as yellow and white immature starters shown separately; mature starters: B0-B5; immature starters: W, Y. Shannon index (C) and observed OTUs numbers (D) of starters divided in two groups (mature and immature).

3 Supplementary Tables

Table S1. Sequencing data of each sample.

Table S2. Spearman's correlation between mature OTUs and physiochemical properties (only show results with adjusted P value < 0.05).

Table S3. Wilcoxon rank-sum test of relative abundances of 21 OTUs in white and yellow starters, including 7 Leave-One-Out Cross Validation markers.

Table S4. Importance of OTUs selected in 54 random forest classifiers of leave-one-out cross validation.

Reference

Ministry of Industry and Information Technology of China (2011). "General methods of analysis for Daqu (QB/T 4257-2011)". (Beijing, China: The Standards Press of China).

Shandong Bureau of Quality and Technical Supervision (2009). "General Specifications of Daqu (DB37/T 1231-2009)". (China).

Standardization Administration of the People’s Republic of China (SAC) (2008). "Determination of reducing sugar in foods (GB/T 5009.7-2008)". (Beijing, China: The Standards Press of China).
